# Uncertainty in GRACE/GRACE-follow on global ocean mass change estimates due to mis-modeled glacial isostatic adjustment and geocenter motion

**DOI:** 10.1038/s41598-022-10628-8

**Published:** 2022-04-22

**Authors:** Jae-Seung Kim, Ki-Weon Seo, Jianli Chen, Clark Wilson

**Affiliations:** 1 Department of Earth Science Education, Seoul National University, Seoul, 08826 Republic of Korea; 2 Center for Educational Research, Seoul National University, Seoul, 08826 Republic of Korea; 3 Center for Space Research, University of Texas at Austin, Austin, TX 78759 USA; 4 Department of Geological Sciences, Jackson School of Geosciences, University of Texas at Austin, Austin, TX 78712 USA

**Keywords:** Climate sciences, Ocean sciences

## Abstract

Global mean sea level has increased about 3 mm/yr over several decades due to increases in ocean mass and changes in sea water density. Ocean mass, accounting for about two-thirds of the increase, can be directly measured by the Gravity Recovery and Climate Experiment (GRACE) and GRACE Follow-On (GFO) satellites. An independent measure is obtained by combining satellite altimetry (measuring total sea level change) and Argo float data (measuring steric changes associated with sea water density). Many previous studies have reported that the two estimates of global mean ocean mass (GMOM) change are in good agreement within stated confidence intervals. Recently, particularly since 2016, estimates by the two methods have diverged. A partial explanation appears to be a spurious variation in steric sea level data. An additional contributor may be deficiencies in Glacial Isostatic Adjustment (GIA) corrections and degree-1 spherical harmonic (SH) coefficients. We found that erroneous corrections for GIA contaminate GRACE/GFO estimates as time goes forward. Errors in GIA corrections affect degree-1 SH coefficients, and degree-1 errors may also be associated with ocean dynamics. Poor estimates of degree-1 SH coefficients are likely an important source of discrepancies in the two methods of estimating GMOM change.

## Introduction

The Gravity Recovery and Climate Experiment (GRACE) has contributed to major improvements in understanding ice mass balance of major ice sheets (Antarctica and Greenland)^1,2^ and mountain glaciers^3,4^ and changes in terrestrial water storage^4–6^, all important contributors to sea level rise^7^. GRACE has also been able to observe the resulting global mean ocean mass (GMOM) changes of approximately 2.07 mm/yr during 2005–2015^6^. An independent GMOM rate from the difference between satellite Altimeter data and Argo float data (Altimeter-Argo) shows general agreement with the GRACE rate (e.g., Chen et al.^8^; Kim et al.^6^). This difference (Altimeter-Argo) corrects total sea level change (from altimetry) by removing steric sea level change due to density variations (from Argo). The GRACE Follow-On (GFO) mission extends the GRACE time series of surface mass redistribution, although there is a gap (July 2017–April 2018) between GRACE and GFO. The continued growth of the GRACE/GFO time series is expected to provide increasing understanding of contemporary climate change.

In the late phase of the GRACE mission (after August 2016) and into the GFO mission (since June 2018) there have been growing differences between the two GMOM estimates (GRACE/GFO and Altimeter-Argo)^9^. Barnoud et al.^10^ examined both Altimeter and Argo data and showed that uncertainty in Argo salinity data may explain about 40% of the discrepancy, but further study is required to explain the remainder. The discrepancy may be due to multiple causes. First, previous GMOM rates from GRACE were estimated over the global oceans^6,11^ but values may differ over the smaller region sampled by Altimeter-Argo^12^. Second, a GMOM estimate from GRACE/GFO is dependent on choice of Glacial Isostatic Adjustment (GIA) model^6^. Gravity field changes observed by GRACE/GFO include contributions from both contemporary surface mass loads and from GIA, the viscoelastic mantle response to past ice mass changes since the last glaciation. GIA effects are removed using one of a number of available GIA models, but there are differences among models, indicating that their deficiencies likely contribute to GMOM rate uncertainty^10^. Furthermore this uncertainty ought to be assessed over the Altimeter-Argo region, rather than the global oceans, when comparing GRACE and Altimeter-Argo estimates. In this study, we examine uncertainties in GRACE/GFO GMOM and find that varying GIA model choice can yield improved agreement between GMOM estimates from GRACE and Altimeter-Argo. We consider both uncertainty in GIA models over the Altimeter-Argo regions, and in degree-1 terms that must be estimated to obtain an GRACE/GFO GMOM.

## Results

To estimate GMOM using GRACE/GFO, we obtained leakage-corrected terrestrial mass loads using forward modeling^13^ (FM) and subsequently estimated ocean mass redistribution conforming to the shape of geoid considering self-attraction and loading (SAL)^14^. The data are CSR RL06 GRACE/GFO spherical harmonic (SH) coefficients with $\Delta$ C_20_ and $\Delta$ C_30_ replaced by SLR values^15,16^. GIA signals were removed using the model of Peltier et al.^17^, but we consider other GIA models^18–20^ to understand the consequences of model choice. We applied 300 km Gaussian smoothing to the SH coefficients but no decorrelation filter^21^. A decorrelation filter suppresses north–south noise patterns (longitudinal stripes) in GRACE/GFO data, but is also likely to attenuate longitudinally oriented signals (e.g., ice mass loss along Greenland coast lines). Using the smoothed SH coefficients, we estimated leakage corrected mass fields via FM, and then applied SAL to determine ocean water distribution. FM iteratively updates mass changes on land and the negative of this is used to compute changes in mean ocean mass (see Chen et al.^13^ for the detail of the FM method). SAL is then used to compute the spatial distribution of ocean mass changes^22^. At each iteration, degree-1 coefficients were generated, and then removed so that the converged FM solution does not include them. Then, using the converged FM solution, we estimated degree-1 coefficients using equations (1)–(6) in Supplementary Information. Our method to estimate degree-1 terms differs from previous studies in that leakage effects were corrected by FM rather than by using an empirical ocean buffer zone^23^ and ocean mass distribution was realized using SAL rather than by assuming a uniform sea level change^24^.

GRACE/GFO GMOM changes were obtained from the SAL estimation, and we refer to the estimate as GMOM^SAL^. Ocean dynamics and atmospheric loading signals, including the inverted barometer effect, were removed in GRACE processing using GRACE/GFO dealiasing fields, so these fields were added to GMOM^SAL^^25^. Global mean atmospheric mass change was corrected to ensure global mass conservation between the terrestrial surface and atmosphere^8^. The correction mainly affects seasonal GMOM^SAL^ variations.

Altimeter-Argo GMOM is based on data from various altimetry data centers that provide monthly gridded total sea level change (including steric effects and ocean mass changes). These include observations from a number of altimetry satellites. We used ensemble means of two datasets: Copernicus Marine Environment Monitoring Service (CMEMS) and Commonwealth Scientific and Industrial Research Organization (CSIRO). Ocean bottom deformation associated with GIA was corrected using the same GIA model^17^ as in GRACE/GFO data processing. Further ocean bottom deformation resulting from contemporary surface mass load redistribution was estimated using leakage corrected GRACE/GFO mass fields over land and associated SAL water distribution over the oceans as discussed above. Altimetry observations were corrected for this^26^, unlike previous studies^6,7,10,27^.

GMOM is estimated from altimetry observations by subtracting steric sea level changes. Steric change is caused by temperature (T) and salinity (S) variations. Prior to about the year 2000, T and S were obtained from shipboard measurements (e.g., XBT) with limited spatial and depth sampling (typically to depths near 700 m). With the advent of the Argo float network, spatial coverage is now near-global and depths extend to 2000 m. Several data centers provide gridded T and S estimates from Argo. We used results from three groups: Scripps Institution of Oceanography^28^, International Pacific Research Center (IPRC) and Japan Agency for Marine-Earth Science and Technology (JAMSTEC)^29^. We used ensemble means of the three data sets which should suppress some errors^7,27^. There may also be steric variations in the abyssal oceans^30–32^, not sampled by Argo. This contribution to global mean sea level is highly uncertain. Estimates for the past decade include −0.13 ± 0.34 mm/yr^31^ and 0.29 ± 0.21 mm/yr^32^. The deep ocean steric effect is not considered in this study but is potentially a source of differences between GRACE/GFO and Altimeter-Argo estimates. Our Altimeter-Argo GMOM estimate is denoted as GMOM^A-A^ hereafter.

Argo float spatial sampling became nearly global since about 2005, so we estimate GMOM^A-A^ after 2005. Neither Altimetry nor Argo floats sample high latitude oceans, nor are all coastal areas sampled. The Altimeter-Argo ocean region (see Fig. S1), is used to estimate GMOM for both Altimeter-Argo (GMOM^A-A^) and GRACE/GFO (GMOM^SAL^). Previous studies (e.g.,Kim et al.^6^,Chen et al.^8^) did not consider a common region when forming the two estimates of GMOM.

**Figure 1:**
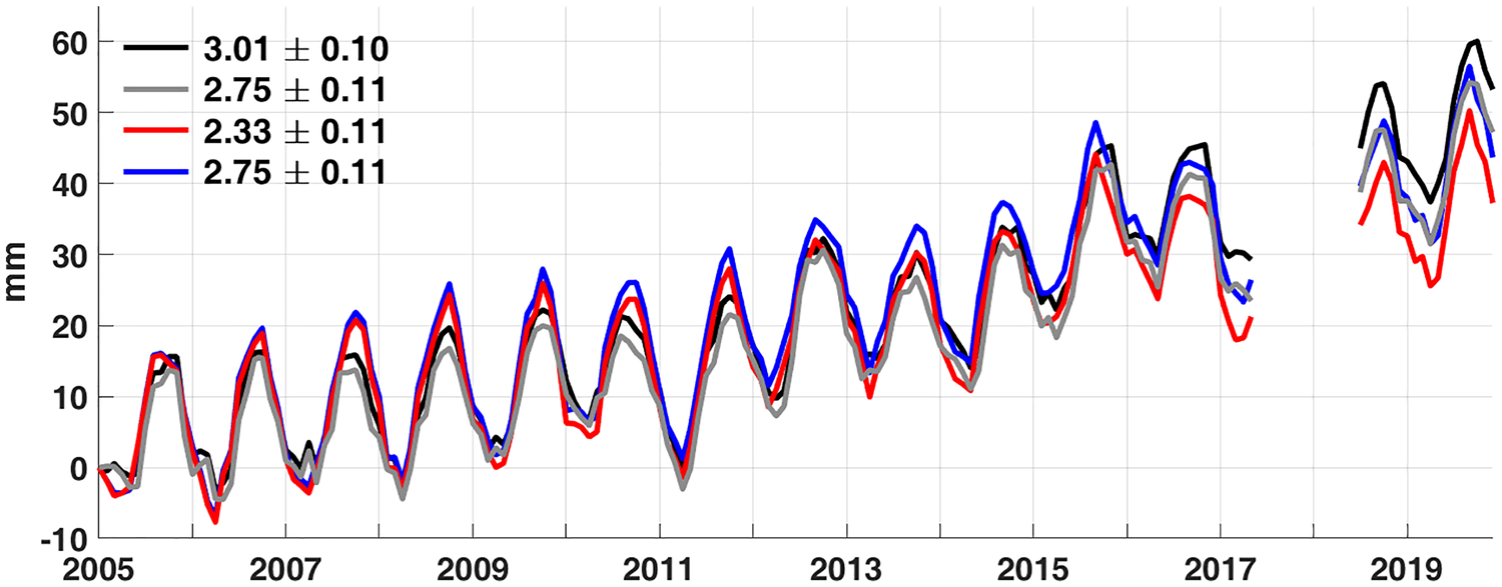
GMOM^SAL^ from GRACE/GFO (red and blue) and GMOM^A-A^ from Altimeter-Argo (black and gray). The black line is based upon an ensemble average of Argo data including both thermosteric and halosteric contributions. The gray line is similar to the black but only includes a thermosteric contribution. Red and blue lines were based on GIA models of Peltier et al.^17^ and Caron et al.^20^, respectively. Numbers are associated linear trends with 95% confidence intervals.

We compare GMOM^SAL^ and GMOM^A-A^ in Fig. 1. As examined previously^9,10^, the GMOM^A-A^ trend in black (Altimeter-Argo) and GMOM^SAL^ trend in red differ significantly after August 2016. However, evident differences between the two are already found during Northern Hemisphere Spring starting in 2013. The trend from the black line is 3.01 mm/yr, which is 0.11 mm/yr larger than the previous estimate for the same period^10^. This is mainly because the previous study did not consider seafloor load deformation. The correction for seafloor deformation is 0.08 mm/yr for the global oceans and 0.17 mm/yr for the Altimeter-Argo region.

We can compare GMOM^SAL^ with Mascon solutions from CSR^33^ and JPL^34^ over the same Altimeter-Argo ocean region. Table 1 shows the three different GMOM trends from our estimate, based on CSR RL06 SH coefficients, and CSR and JPL Mascons. Our estimate (2.33 ± 0.11 mm/yr) is close to Mascon values, particularly to CSR (2.24 ± 0.12 mm/yr). Barnoud et al.^10^ also estimated a GMOM trend from the same CSR Mascon solutions, finding a value of 2.08 mm/yr, somewhat smaller than our estimate. The difference is due to the ocean regions used. Like Barnoud et al.^10^ we excluded latitudes above 66° but here we also exclude coastal areas not sampled by Altimeter-Argo (see Fig. S1). We obtained the same value (2.08 ± 0.09 mm/yr) by including these coastal regions.

**Table 1:** GMOM comparisons between this study and Mascons.

	This study	CSR	JPL
66°N–66°S	2.29 ± 0.10 (GMOM^SAL^)	2.08 ± 0.09	2.19 ± 0.08
Altimetry & Argo coverage	2.33 ± 0.11 (GMOM^SAL^)	2.24 ± 0.12	2.17 ± 0.11

Barnoud et al.^10^ showed that spurious trends in halosteric sea level from Argo data partly explained the discrepancy between GRACE/GFO and Altimeter-Argo estimates (called here GMOM^SAL^ and GMOM^A-A^) after 2016. To test this, we estimated GMOM^A-A^ using ensemble-averaged thermosteric-only sea level (gray line in Fig. 1). The trend difference between gray (2.75 mm/yr) and red (2.33 mm/yr) lines is smaller than between black and red lines.

**Figure 2:**
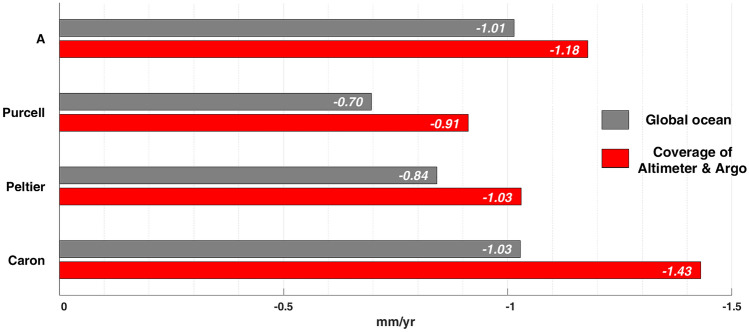
Apparent ocean mass rates due to GIA from GIA models of A et al.^18^,Purcell et al.^19^,Peltier et al.^17^, and Caron et al.^20^. Gray and red bars represent apparent water mass loads over global oceans and coverage of Altimeter and Argo, respectively.

The remaining trend difference between GMOM^A-A^ and GMOM^SAL^ is likely due to deficiencies in GIA models. We estimated apparent water mass loads using the four GIA models of A et al.^18^, Purcell et al.^19^, Caron et al.^20^ and Peltier et al.^17^. This GIA mass load change should be removed from GRACE/GFO data to estimate GMOM^SAL^. However, there are differences among the four GIA model estimates (denoted by OM^A^, OM^Purcell^, OM^Caron^ and OM^Peltier^ hereafter). The four models provide GIA mass changes over the global oceans shown by gray bars in Fig. 2. OM^Caron^ is the largest, −1.03 mm/yr, about −0.19 mm/yr larger than OM^Peltier^. In general, differences among GIA models over the global oceans are less than remaining difference between gray (2.75 mm/yr) and red (2.33 mm/yr) lines in Fig. 1. However, over the Altimeter-Argo ocean region (red bars in Fig. 2), differences among models are greater. In particular, OM^Caron^ yields −1.43 mm/yr, about −0.40 mm/yr larger than over the global oceans. This is mainly because large values in OM^Caron^ over the southern oceans are excluded in the Atlimeter-Argo region (Fig. S1). As a result, GRACE/GFO estimates (using the GIA model of Caron et al.^20^) show an increased GMOM^SAL^ rate over the Altimeter-Argo ocean region, 2.75 mm/yr (blue line in Fig. 1), very near that of GMOM^A-A^ with thermosteric-only corrections (gray line in Fig. 1).

However trend differences between GMOM^SAL^ and GMOM^A-A^ depend on the selected portion of the time series. For example, trends of blue and black lines for 2005–2015 are 2.72 ± 0.14 mm/yr and 2.51 ± 0.16 mm/yr, respectively. For this period, the GMOM^SAL^ rate (2.42 ± 0.14 mm/yr) using the GIA model of A et al.^18^ is closer to GMOM^A-A^ (2.51 ± 0.16 mm/yr). Varying trend differences between GMOM^SAL^ and GMOM^A-A^ are likely assocated with data uncertainties. This suggests that it is difficult to judge the best GIA model choice among the four by comparing GMOM^SAL^ and GMOM^A-A^.

**Figure 3:**
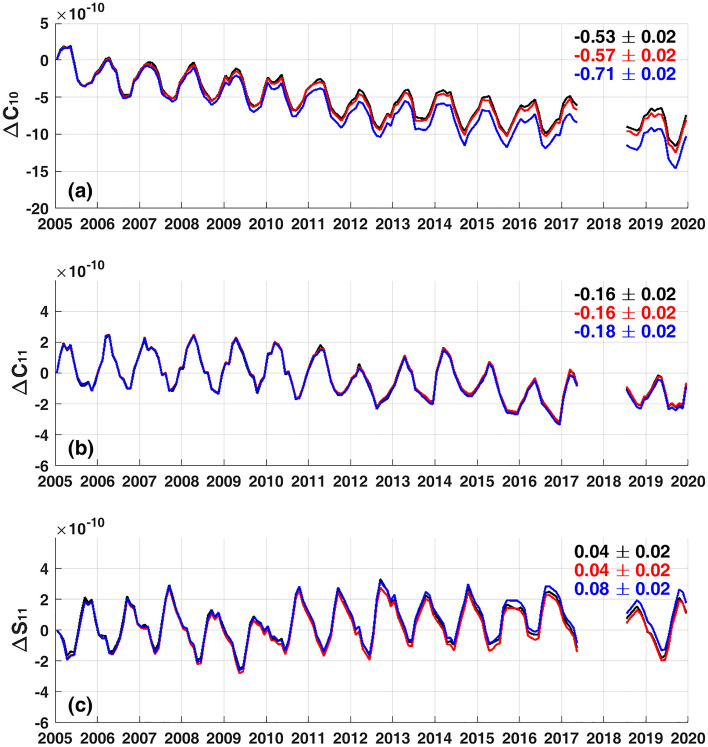
Degree-1 SH Coefficients. Gray lines are from TN13, and red and blue lines are estimated SH coefficients after GIA correction with Peltier et al.^17^ and Caron et al.^20^, respectively. Numbers in each panel are linear trends with associated 95% confidence intervals.

The trend difference between blue and red lines in Fig. 1 (GMOM^SAL^ based respectively on the GIA models of Caron et al.^20^ and Peltier et al.^17^) is −0.43 mm/yr. Different estimates of SH degree-1 terms explain about −0.12 mm/yr of the trend difference, −0.43 mm/yr. Figure 3 shows estimated SH degree-1 terms from GRACE/GFO mass fields using GIA models of Caron et al.^20^ (blue) and Peltier et al.^17^ (red). There are evident trend differences in the two estimates, particularly in $\Delta$ C_10_. Degree-1 terms from Sun et al. (TN13)^23^ (black) are close to the red lines because the same GIA model of Peltier et al.^17^ was used. Because SH degree-1 terms depend upon the adopted GIA model, TN13 can be used when adopting the Peltier model, but must be estimated for other models.

In summary, variations among GIA models over the Altimeter-Argo region are large enough to explain differences in GMOM^SAL^ and GMOM^A-A^ estimates, and associated degree-1 SH coefficients, are also affected by GIA model and play an important role in these differences.

## Discussion

Even though GIA models exclude SH degree-1 terms, residual GIA effects (GIA model errors evident from differences among models) contaminate degree-1 estimates derived from other harmonics via equations (1)–(6) in Supplementary Information. Similarly, residual ocean dynamics (ROD) in GRACE/GFO after the correction of ocean dynamic effects during GRACE processing may contribute to errors in degree-1 terms. ROD should have a small effect when averaged over a large area, including the global oceans or the Altimeter-Argo region, but they might still contaminate estimates of degree-1 terms with related effects on GMOM estimates. We can estimate sensitivity to ROD using synthetic GRACE-like data (see “Method” and Fig. S2), along with ocean dynamics fields used in GRACE processing (GAD) and ocean bottom pressure data that provide a measure of ROD effects.

Here we examine two measures of ROD effects. One is the difference between GAD for CSR RL06 and that for CSR RL05 (hereafter ROD1), and the other is the difference between CSR RL06 GAD and bottom pressure data from the German contribution to Estimating the Circulation and Climate of the Ocean (GECCO2)^35^ (hereafter ROD2) (Fig. S3). ROD2 is much larger than ROD1, so the two choices are useful to understand possible ROD contamination in degree-1 terms. Long-term ROD variations, particularly from GECCO2, cause a spurious trend in global mean ROD. To suppress this, ROD values at grid points were adjusted to have zero global mean every month. The same 300 km Gaussian smoothing was applied to the synthetic data, as for GRACE/GFO.

Using the synthetic data (see “Method”) with either ROD1 or ROD2, we estimated degree-1 terms and compared them with known true values for the synthetic data. Because the negative of total terrestrial mass change and SAL were used to compute ocean mass loads, our ocean mass fields do not include ROD. However, when estimating degree-1 terms, ROD is inevitably included (see Supplementary Information and Fig. S3) and would affect GMOM estimation.

**Figure 4:**
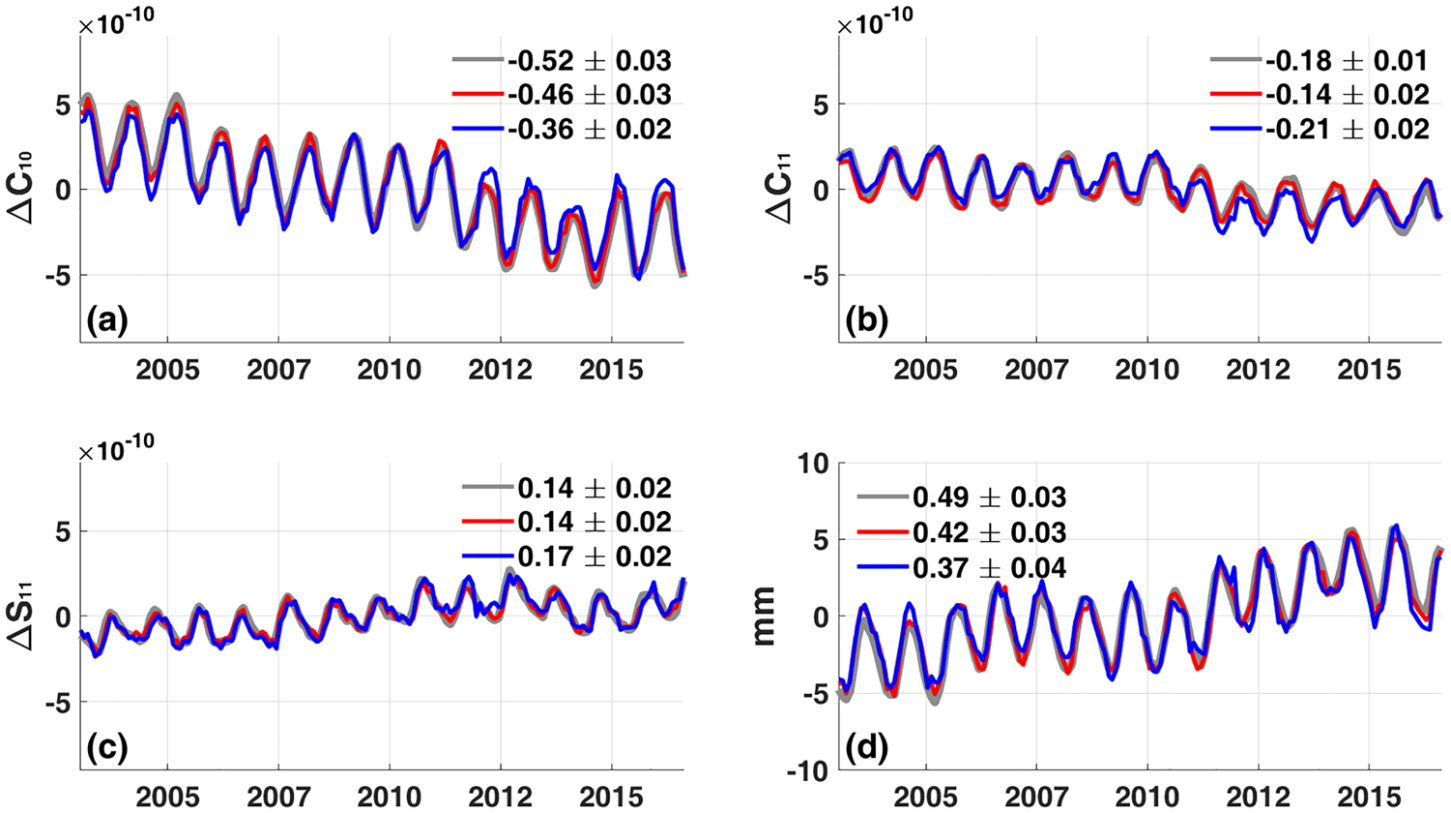
Estimates of degree-1 terms and their effects on GMOM from synthetic GRACE-like data. (**a**)–(**c**) Gray lines are true degree-1 SH coefficients. Red and blue lines are estimated degree-1 SH coefficients from synthetic GRACE-like synthetic data including ROD1 and ROD2, respectively. (**d**) Gray line is true GMOM only considering degree-1 terms over Altimeter- Argo regions. Red and blue lines are GMOM using estimated degree-1 SH coefficients with ROD1 and ROD2, respectively. Numbers in each panel are linear trends with associated 95% confidence intervals.

Figure 4 shows synthetic true degree-1 terms and estimates contaminated by estimated ROD effects (ROD1 and ROD2). Gray lines in Fig. 4(a)–(c) are true variations and red lines are estimated with ROD1. Because of the small magnitude of ROD1, gray and red lines are almost identical. The larger magnitude ROD2 (blue lines), shows much larger differences between true and estimated degree-1 SH coefficients. Figure 4(d) shows the degree-1 contributions to GMOM. ROD1 (red) is close to true (gray), while ROD2 (blue) causes much larger differences. The trend difference between blue and gray is 0.12 mm/yr, mostly due to the trend difference in $\Delta$ C_10_. In practice it is difficult to assess the importance of ROD, due to sparse in-situ observations of ocean bottom pressure. However this experiment shows that it may be possibly a source of significant error in degree-1 estimates.

## Conclusions

Estimates of global mean ocean mass (GMOM) variations are provided by observations of GRACE/GFO and Altimeter-Argo. The two GMOM estimates are in good agreement during the early period (2005–2015), but differences increase beyond this period. A spurious halosteric sea level trend was suggested as the possible cause of the disagreement, but only explained the abrupt difference after August 2016.

The remaining difference is possibly due to GIA model error. GMOM trends from GRACE/GFO are heavily affected by GIA model choice. Four GIA models yield variable rates: 2.75 ± 0.11 mm/yr (model of Caron et al.^20^) for January 2005 to December 2019, is close to the Altimeter-Argo trend (using thermosteric-only sea level changes) of 2.75 ± 0.11 mm/yr for the same period. The trend associated with the model of Peltier et al.^17^ is much smaller, 2.33 ± 0.11 mm/yr. Different estimates of degree-1 terms partly explain the GMOM trend differences. Even though GIA models do not include degree-1 SH coefficients, they alter other GRACE/GFO SH coefficients used to estimate degree-1 terms. Degree-1 uncertainty may also be caused by residual ocean dynamic (ROD) effects, as indicated by experiments with synthetic GRACE-like data. Sparse observations of ocean bottom pressure make it difficult to be more precise.

GFO continues to extend the time series begun by GRACE, and further understanding of contemporary climate change, including ice mass variations, should follow in the future. However, this study shows that further progress is needed in development of GIA models. These models estimate SH coefficient rates of change, so associated errors will grow as the time span increases. Current methods of estimating degree-1 SH terms are also a limitation in estimates of secular trends in surface mass distribution.

## Method

### Synthetic GRACE data

Synthetic data were used to understand uncertainty in estimates of degree-1 SH coefficients. We used synthetic GRACE-like data to compare estimated degree-1 coefficients with true values from the synthetic data. Synthetic GRACE-like data were computed using surface mass fields from numerical climate models and other sources in the literature. The global land data assimilation system (GLDAS)^36^ was used to represent terrestrial water storage change. Ice mass variations in Greenland and Antarctica were taken from Kim et al.^6^, and mountain glaciers from Zemp et al.^3^. Figure S2(a) shows an example of synthetic surface mass loads. We estimated GRACE errors as the difference between GRACE and Gaussian smoothed GRACE solutions^37,38^. This error estimate would also include residual surface mass signals. To remove these residual signals, we employed an empirical orthogonal functions (EOF) method which decomposes spatially and temporally correlated fields^38^. The EOF method should distinguish and allow separation of residual signals within the difference between GRACE and Gaussian smoothed GRACE data. Residual surface mass load signals ought to be both spatially and temporally distinct from actual errors. Figure S2(b) shows an example of GRACE error estimated here and Fig. S2(c) shows synthetic GRACE-like data (sum of synthetic GRACE-like signal (Fig. S2(a)) and errors (Fig. S2(b))).

## Supplementary Information

Supplementary Information.
