# Supplementary material for: Uncertainty in GRACE/GRACE-follow on global ocean mass change estimates due to mis-modeled glacial isostatic adjustment and geocenter motion
Source: Sci Rep. 2022 Apr 22;12:6617. doi: 10.1038/s41598-022-10628-8 (PMC9033860; doi:10.1038/s41598-022-10628-8)
Supplement: Supplementary file 1 — Supplementary Information. [file 41598_2022_10628_MOESM1_ESM.docx]

**Supplementary Information**

**Estimation of Degree-1 SH coefficients**
 A vector $\bar{X}$ includes degree-1 SH coefficients and additional cosine SH coefficient of degree *i*and order *j* to be estimated, i.e. $\bar{X}= \left[ C_{10} C_{11} S_{11} \right]^{T}$. $\bar{X}$ satisfies the linear equation ^1^

| $\bar{O}= \bar{I}\bar{X}+ \bar{G}$ | (1) |
| --- | --- |

where $\bar{O}$ represents SH coefficients only for ocean mass distribution

| $\bar{O}={[C_{10}^{ocean} C_{11}^{ocean} S_{11}^{ocean}]}^{T}$ | (2) |
| --- | --- |

The $\bar{I}$ matrix is

| $\bar{I}=\frac{1}{4\pi} \int\bar{U}\bar{U}^{T} \vartheta(\theta, \phi) d\Omega$ | (3) |
| --- | --- |

where $\Omega$ is a position vector in spherical coordinates (latitude ($\phi$) and longitude ($\theta$)). $\bar{U}$ is consist of

| $\bar{U}= \left[ U_{10C} U_{11C} U_{11S} \right]^{T}$ | (4) |
| --- | --- |

, and the element of $\bar{U}$ is

| $U_{lm\psi}=\tilde{P}_{lm}\left( \cos\theta\right)\left\{ \begin{aligned} \cos\left( m\phi\right) ( \psi=C) \\ \sin\left( m\phi\right) ( \psi=S) \end{aligned} \right.$ | (5) |
| --- | --- |

Finally, $\bar{G}$ matrix is

| $G_{lm\psi}= \frac{1}{4\pi}\int U_{lm\psi}\vartheta\left( \theta, \phi\right)\sum_{l^{'}=2}^{\infty} \sum_{m^{'}=0}^{l^{'}} \tilde{P}_{l^{'}m^{'}}\left( \cos\theta\right)\left\{ C_{l^{'}m^{'}}\cos\left( m^{'}\phi\right)+S_{l^{'}m^{'}}\sin\left( m^{'}\phi\right) \right\} d\Omega$ | (6) |
| --- | --- |

We can obtain those SH coefficients by solving equation (1) iteratively updating $\bar{O}$ until $\bar{X}$ converges.


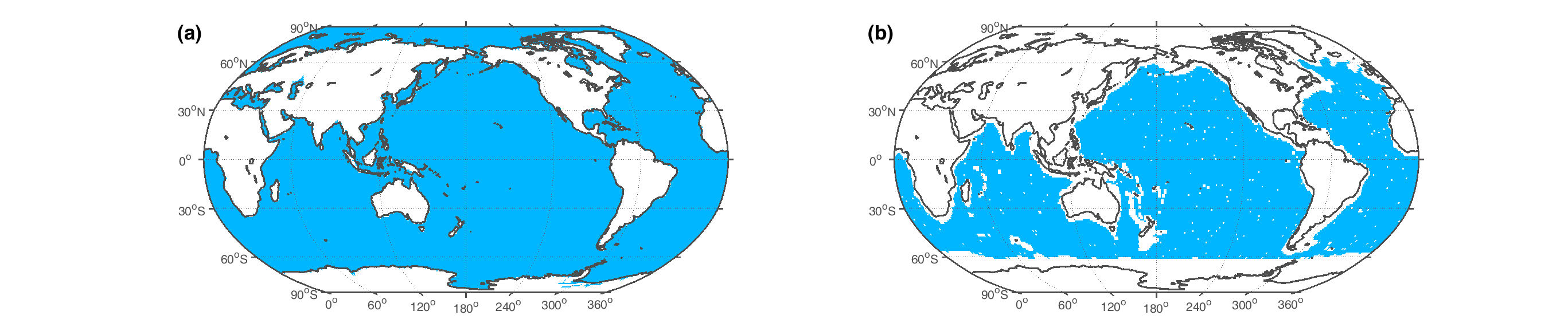


Figure S1. Considered Ocean Coverage. GMOM is estimated over two different areas, (a) global oceans and (b) Altimeter and Argo ocean region. This figure is created with MATLAB R2020a (https://kr.mathworks.com/help/releases/R2020a/relnotes/).


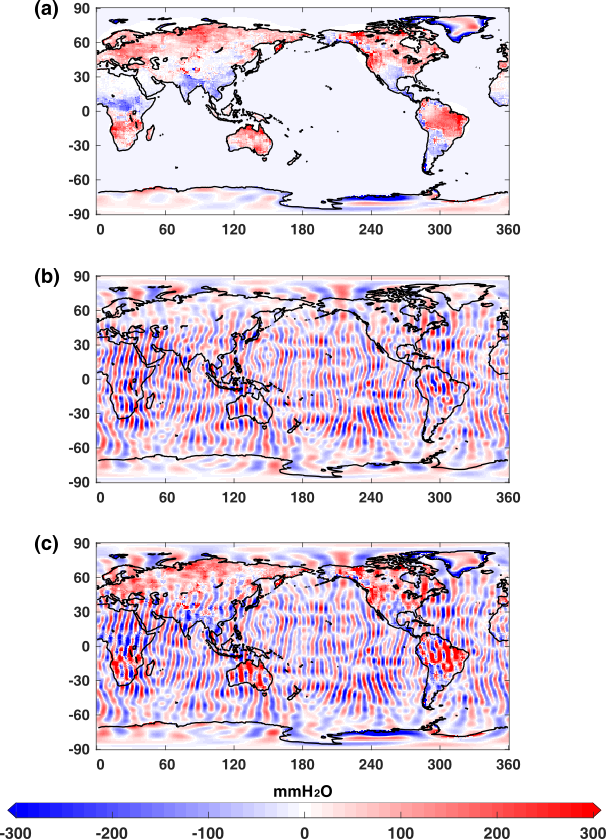


Figure S2. Synthetic GRACE-like Data. Synthetic data includes (a) surface mass loads and (b) error. The surface mass loads consist of variations of terrestrial water storage, ice mass over mountain glaciers and ice sheets, and ocean mass changes derived from terrestrial water and ice mass changes including SAL effect. (c) is synthetic GRACE-like data (sum of (a) and (b)). This figure is created with MATLAB R2020a (https://kr.mathworks.com/help/releases/R2020a/relnotes/).

**Residual ocean dynamic effect in degree-1 SH coefficients** This section examines how residual ocean dynamic (ROD) effects may contaminate degree-1 estimates. Earth surface mass loads ($\sigma$) can be divided into land ($\sigma_{land}$) and ocean ($\sigma_{land}$) parts including ROD over land ($R_{land}$) and oceans ($R_{ocean}$):

| $\sigma\left( \Omega\right)= \sigma_{land}\left( \Omega\right)+\sigma_{ocean}\left( \Omega\right)+R_{land}\left( \Omega\right)+R_{ocean}\left( \Omega\right)$ | (7) |
| --- | --- |

where, $\Omega$ is a position vector in spherical coordinates (e.g., latitude and longitude). Equation (7) can be represented by SH coefficients of $\sigma$ and $R$:

| $\sigma\left( \Omega\right)= \sum_{l=1}^{N} \sum_{m=0}^{l} \tilde{P}_{lm}\left( cos\theta\right)\{\left( C_{lm}^{\sigma_{land}}+ C_{lm}^{\sigma_{ocean}}+C_{lm}^{R_{ocean}} \right)\cos\left( m\phi\right)$ $+ \left( S_{lm}^{\sigma_{land}}+ S_{lm}^{\sigma_{ocean}}+ S_{lm}^{R_{ocean}} \right)\sin\left( m\phi\right)$} (8) $+\sum_{l=1}^{N} \sum_{m=0}^{l} \tilde{P}_{lm}\left( cos\theta\right)\left( C_{lm}^{R_{land}}\cos\left( m\phi\right)+S_{lm}^{R_{land}}\sin\left( m\phi\right) \right)$ |  |
| --- | --- |

where $\tilde{P}_{lm}$ are normalized associated Legendre functions, and $\left( l,m \right)$ are (degree, order) of SH. ($C_{lm}^{\sigma_{land}}$,$S_{lm}^{\sigma_{land}}$) are SH coefficients of surface mass loads ($\sigma$) for land, and ($C_{lm}^{\sigma_{ocean}}$,$S_{lm}^{\sigma_{ocean}}$) for oceans. Similarly, ($C_{lm}^{R_{land}}$,$S_{lm}^{R_{land}}$) and ($C_{lm}^{R_{ocean}}$,$S_{lm}^{R_{ocean}}$) are SH coefficients of ROD for land and oceans, respectively. When the maximum degree, N, is sufficiently large, the sum of all contribution from ($C_{lm}^{R_{land}}$,$S_{lm}^{R_{land}}$) in Equation (8) is close to zero. Because we aim to estimate degree-1 SH coefficients with higher degree SH coefficients, it is necessary to remove degree 1 terms,

| $\sigma\left( \Omega\right)- \sum_{l=1}^{1} \sum_{m=0}^{l} \tilde{P}_{lm}\left( cos\theta\right)\{\left( C_{lm}^{\sigma_{land}}+ C_{lm}^{\sigma_{ocean}}+ C_{lm}^{R_{land}}+ C_{lm}^{R_{ocean}} \right)\cos\left( m\phi\right)$+ $\left( S_{lm}^{\sigma_{land}}+ S_{lm}^{\sigma_{ocean}}+ S_{lm}^{R_{land}}+S_{lm}^{R_{ocean}} \right)\sin\left( m\phi\right)\}$ $= \sum_{l=2}^{N} \sum_{m=0}^{l} \tilde{P}_{lm}\left( cos\theta\right)\{\left( C_{lm}^{\sigma_{land}}+ C_{lm}^{\sigma_{ocean}}+C_{lm}^{R_{ocean}} \right)\cos\left( m\phi\right)$ $+ \left( S_{lm}^{\sigma_{land}}+ S_{lm}^{\sigma_{ocean}}+ S_{lm}^{R_{ocean}} \right)\sin\left( m\phi\right)$} |  |
| --- | --- |
| $+\sum_{l=0}^{2} \sum_{m=0}^{l} \tilde{P}_{lm}\left( cos\theta\right)\left( C_{lm}^{R_{land}}\cos\left( m\phi\right)+S_{lm}^{R_{land}}\sin\left( m\phi\right) \right)$ | (9) |

Equation (9) represents surface mass load and ROD realized by SH coefficients from degree 2 to N as observed by GRACE/GFO. These are used to estimate leakage corrected terrestrial mass load via FM and its subsequent distribution over the oceans using Self Attraction and Loading (SAL). In this case, $R_{land}$associated with SH degrees 2 and higher is not zero, and as a result, this ROD effect is included as an apparent terrestrial mass loads (Fig. S3 (c) and (d)). This ROD contamination of terrestrial surface mass loads may further affect leakage corrected mass fields and SAL, and eventually degree-1 SH coefficients.


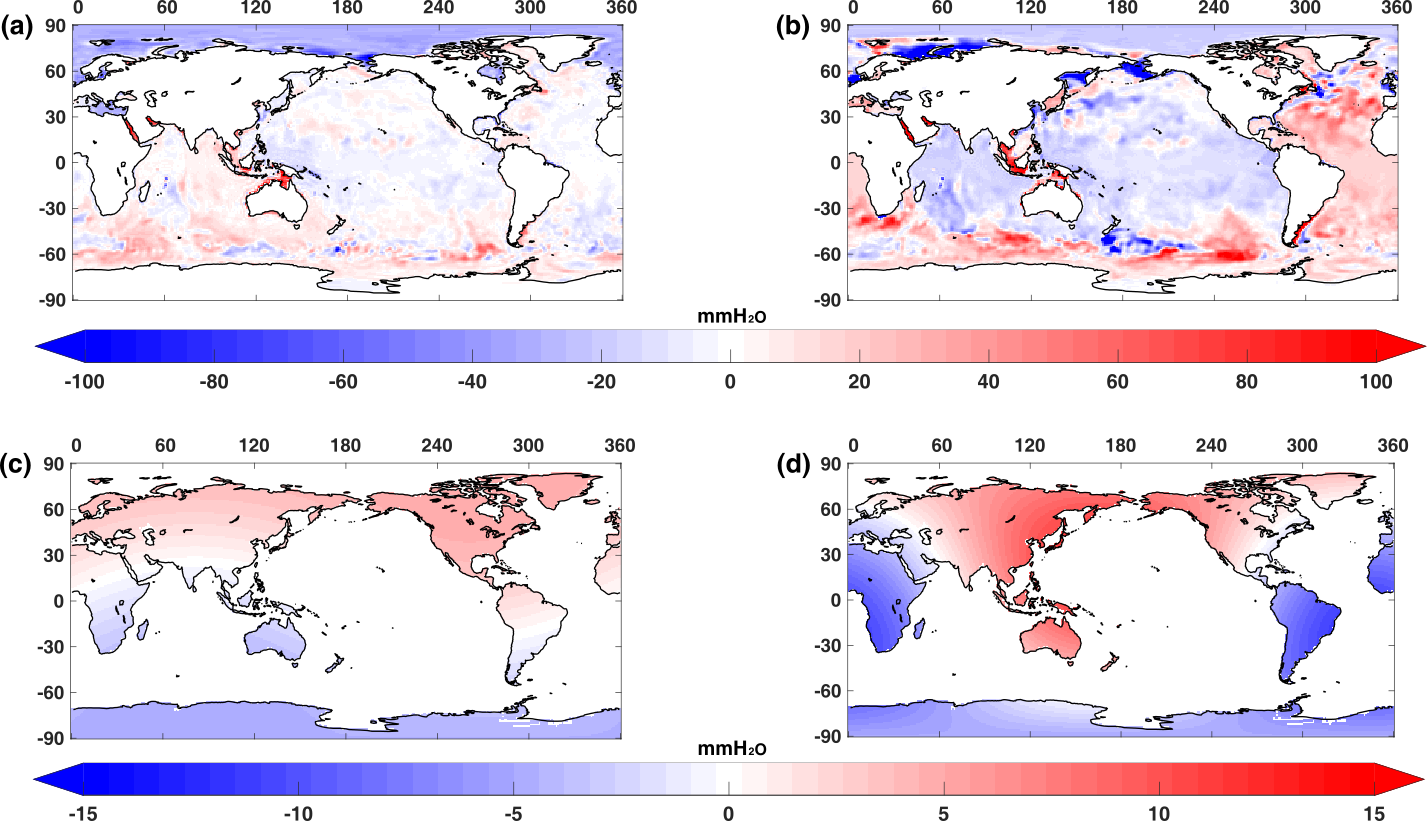


Figure S3. Synthetic residual ocean dynamic (ROD) effect. (a) and (b) are ROD1 (GAD RL06-GAD RL05) and ROD2 (GAD RL06-GECCO2), respectively. (c) and (d) are apparent terrestrial mass fields associated with ROD1 and ROD2, respectively. This figure is created with MATLAB R2020a (https://kr.mathworks.com/help/releases/R2020a/relnotes/)

**Reference**

1 Swenson, S., Chambers, D. & Wahr, J. Estimating geocenter variations from a combination of GRACE and ocean model output. *J. Geophys. Res. Solid Earth* **113**, B08410, doi:10.1029/2007jb005338 (2008).
